# Associations Between Lifestyle Factors and Reduced Kidney Function in US Older Adults: NHANES 1999–2016

**DOI:** 10.3389/ijph.2021.1603966

**Published:** 2021-07-15

**Authors:** Horng-Jinh Chang, Kuan-Reng Lin, Meng-Te Lin, Junn-Liang Chang

**Affiliations:** ^1^ Department of Management Sciences, Tamkang University, Taiwan; ^2^ Department of Pathology and Laboratory Medicine, Taoyuan Armed Forces General Hospital, Taoyuan, Taiwan; ^3^ Biomedical Engineering Department, Ming Chuan University, Taoyuan, Taiwan

**Keywords:** chronic kidney disease, estimated glomerular filtration rate, exercise, kidney function, risk factors

## Abstract

**Objective:** This study aimed to evaluate the associations between lifestyle factors and the estimated glomerular filtration rate (eGFR) levels in older adults by analyzing the United States National Health and Nutrition Examination Survey data (1999–2016).

**Methods:** A total of 10,052 eligible participants were divided into two groups: reduced eGFR group (eGFR < 60 ml/min/1.73 m^2^) and normal group (eGFR ≥ 60 ml/min/1.73 m^2^). The primary factors were physical activity, alcohol consumption, smoking, and comorbidities.

**Results:** Multivariable analysis revealed that older age, proteinuria, cardiovascular disease, diabetes, hyperuricemia, and hypertension were significantly associated with higher odds of reduced kidney function. Sufficient physical activity, current alcohol consumption, and being a current smoker were significantly associated with lower odds of reduced kidney function. However, subgroup analysis by sex revealed that the effects of proteinuria, current alcohol consumption, and sufficient physical activity were sex-specific.

**Conclusion:** Several risk and beneficial factors for reduced kidney function in adults aged 65 and above in the United States were identified, but some of them might be sex-specific. Further studies are warranted to confirm these findings in other populations and countries.

## Introduction

Populations around the world are aging as a result of healthcare advances that have reduced mortality among youth and increased longevity [1]. Accompanying the increasing older adult population, however, are the inevitable age-associated declines in organ function and biological systems, including changes in the heart and vascular system, the lungs and respiratory system, central nervous system, and cognitive function [2]. Aging itself is suggested to be a risk factor for cardiovascular disease [3]. The decline in kidney function, rather than directly associated with aging, appears to result from age-related changes in the vasculature and cardiovascular risk factors, as well as from genetic factors [4, 5]. That is, the increased prevalence of cardiovascular disease influences the prevalence of chronic kidney disease (CKD), although it is not known to what extent compared to the effects of aging such as renal structural changes [1]. CKD is reported to affect 47 million (14.8%) adults in the United States (US) alone [6]; the prevalence in the global population is 11–13% [7].

CKD is diagnosed when abnormalities of kidney function or structure have been noted for 3 months or more, including an estimated glomerular filtration rate (eGFR) less than 60 ml per minute per 1.73 m^2^ along with the presence of albuminuria, abnormal urine sediment, abnormal renal imaging findings, serum electrolytes, or acid–base derangements [8]. eGFR is a primary parameter for evaluating kidney function, making it a reliable biological marker for CKD and for monitoring fluctuations in kidney function. All individuals with an eGFR of less than 60 ml/min/1.73 m^2^at least twice within an interval of 90 days with or without other markers of kidney damage are said to have CKD. Risk factors for CKD may also be in evidence, including smoking, excessive alcohol consumption, obesity, hypertension, diabetes mellitus, and a history of cardiovascular disease [9].

In our previous cross-sectional study [10], we analyzed cross-sectional data from the Taipei City Elderly Health Examination database to investigate associations between lifestyle factors and reduced kidney function in older adults. We used the eGFR to evaluate changes in kidney function that may show reduced kidney function, but our objective was not to diagnose CKD. This was because cross-sectional studies using a secondary database cannot ensure whether individual participants had an eGFR of less than 60 ml/min/1.73 m^2^ for more than 3 months, reflecting the guidelines for CKD diagnosis as described above. Among the important findings of our earlier study, older adults who had regular or occasional physical exercise had a significantly lower risk of reduced kidney function than those who did not exercise, while risk factors such as smoking, hypertension, obesity, and diabetes were significantly associated with a lower eGFR [10]. To both expand and confirm our previous results, and to further examine the influence of lifestyle factors on kidney function, the present study aimed to evaluate associations between lifestyle factors and reduced kidney function indicated by the eGFR levels in a large, nationwide sample of older adults in the United States.

## Methods

### Study Population

The present study extracted cross-sectional data of older adults from the United States National Health and Nutrition Examination Survey (NHANES) to evaluate the associations between lifestyle factors and eGFR levels. NHANES is a continuous population-based database compiled in 2-year cycles by the National Center for Health Statistics (NCHS) of the United States Centers for Disease Control and Prevention (CDC), and the data are released every 2 years for research purposes. Since all NHANES data are de-identified, data analysis does not require Institutional Review Board approval or a signed informed consent from the participants.

To maximize the sample size, the data of participants from nine 2-year cycles of the NHANES (from 1999–2000 to 2015–2016) were combined and analyzed. The inclusion criteria were non-institutionalized older adults (aged 65 years and older). Participants with missing data for eGFR, physical activity, alcohol consumption, or cigarette smoking were excluded. Values for eGFR were calculated from serum creatinine standardized by isotope dilution mass spectrometry (IDMS) using the Chronic Kidney Disease Epidemiology Collaboration (CKD-EPI) formula [11]. An eGFR less than 60 ml/min/1.73 m^2^ was defined as reduced kidney function; an eGFR ≥ 60 ml/min/1.73 m^2^ was defined as normal kidney function, as previously described [10]. Because cross-sectional data of national surveys do not include longitudinal data and cannot confirm whether participants had eGFR values less than 60 ml/min/1.73 m^2^ for more than 3 months, this study used “reduced kidney function” to describe participants with lower eGFR rather than suggesting CKD.

### Demographic Variables and Body Mass Index

Age, sex, race, education level, and poverty income ratio were extracted from NHANES for all participants and analyzed. Race/ethnicity was self-reported as Mexican American, Other Hispanic, Non-Hispanic White, Non-Hispanic Black, and Other Race/Ethnicity including multiracial. Education level was classified into three levels: with or without high school diploma and higher than high school. The family income-to-poverty ratio ranges from 0 to 5, with greater values indicating a higher income, and it was divided into three levels: <1, 1–3, and >3. Body mass index (BMI) was classified into four categories—underweight (BMI < 18.5 kg/m^2^), normal (BMI = 18.5–24.9 kg/m^2^), overweight (BMI = 25–29.9 kg/m^2^), and obese (BMI > 30 kg/m^2^)—based on the criteria of the World Health Organization (WHO) (https://www.euro.who.int/en/health-topics/disease-prevention/nutrition/a-healthy-lifestyle/body-mass-index-bmi).

### Lifestyle Factors

The lifestyle factors examined in this study included physical activity, alcohol consumption, and cigarette smoking. In the NHANES database, leisure time physical activity was calculated by summing the product of weekly time spent in each activity multiplied by the metabolic equivalent of task (MET) value for that activity. Based on the MET scores, the participants were divided into three categories: inactive (0 MET-min/week), insufficiently active (<750 MET-min/week), and sufficiently active (≥750 MET-min/week), as previously described [12].

The NHANES defined one alcohol-based drink as 12 oz. of beer, 4 oz. of wine, or an ounce of liquor. Participants who did not have at least 12 alcohol-based drinks in the past year or lifetime were classified as non-drinkers. Participants who had at least 12 drinks in their lifetime but not in the past year were classified as former drinkers. Participants who had at least 12 drinks in the past year and reported the number of drinks per week were recognized as current drinkers.

Participants who never had at least 100 cigarettes in their lifetime were classified as non-smokers. Participants who had at least 100 cigarettes but did not smoke now were classified as former smokers. Participants who had at least 100 cigarettes and reported the number of cigarettes per day in the past 30 days were classified as current smokers.

### Clinical Characteristics

Blood levels of fasting glucose, total cholesterol, high-density lipoprotein (HDL), uric acid, blood urea nitrogen (BUN), and creatinine were obtained from the NHANES laboratory data. In addition, proteinuria was defined as urine albumin/creatinine ratio (UACR) ≥30 mg/g.

The self-reported and the laboratory data of NHANES were utilized to identify participants with comorbidities. Cardiovascular diseases (coronary heart disease, angina pectoris, heart attack, stroke, and congestive heart failure) and cancer were defined by responding “yes” to any questions relative to previous diagnosis. Diabetes was defined as being told that they have diabetes or are at risk of diabetes, or taking insulin or antidiabetic medications. Hyperlipidemia was defined as being told that they have higher cholesterol level; are taking cholesterol-lowering medication; and/or having total cholesterol >200 mg/dl, triglyceride >200 mg/dl, or low-density lipoprotein (LDL) > 130 mg/dl. Hyperuricemia was defined as being told that they have hyperuricemia or serum uric acid >7 mg/dl. Hypertension was defined as being told at least twice that they have high blood pressure, are taking antihypertensive medication, and/or having systolic blood pressure ≥ 140 mmHg or diastolic blood pressure ≥ 90 mmHg. Urinary tract stones (renal calculi) were self-reported, but the corresponding NHANES questionnaire was only available from 2007 to 2016.

### Statistical Analysis

Continuous variables are presented as the mean ± standard error (SE), and differences were examined using the complex samples general linear model (CSGLM). Categorical variables are presented as counts and weighted percentages, and differences were tested by the Rao–Scott chi-square test. Interactions between covariates were evaluated. Univariate and multivariable regression models were constructed to identify factors associated with reduced kidney function. Significant interaction terms and significant variables identified in univariate analyses were used to establish the final multivariable models. Sensitivity analysis was performed to confirm the findings of the multivariable analysis, and subgroup analysis by sex was conducted. Special sample weights, stratum, and primary sampling units (PSUs) recommended by the NCHS were all included in the statistical analyses. All statistical analyses were performed using SAS® version 9.4 (Windows NT version, SAS Institute, Inc., Cary, NC, United States). A *p*-value < 0.05 was considered statistically significant.

## Results

**FIGURE 1:**
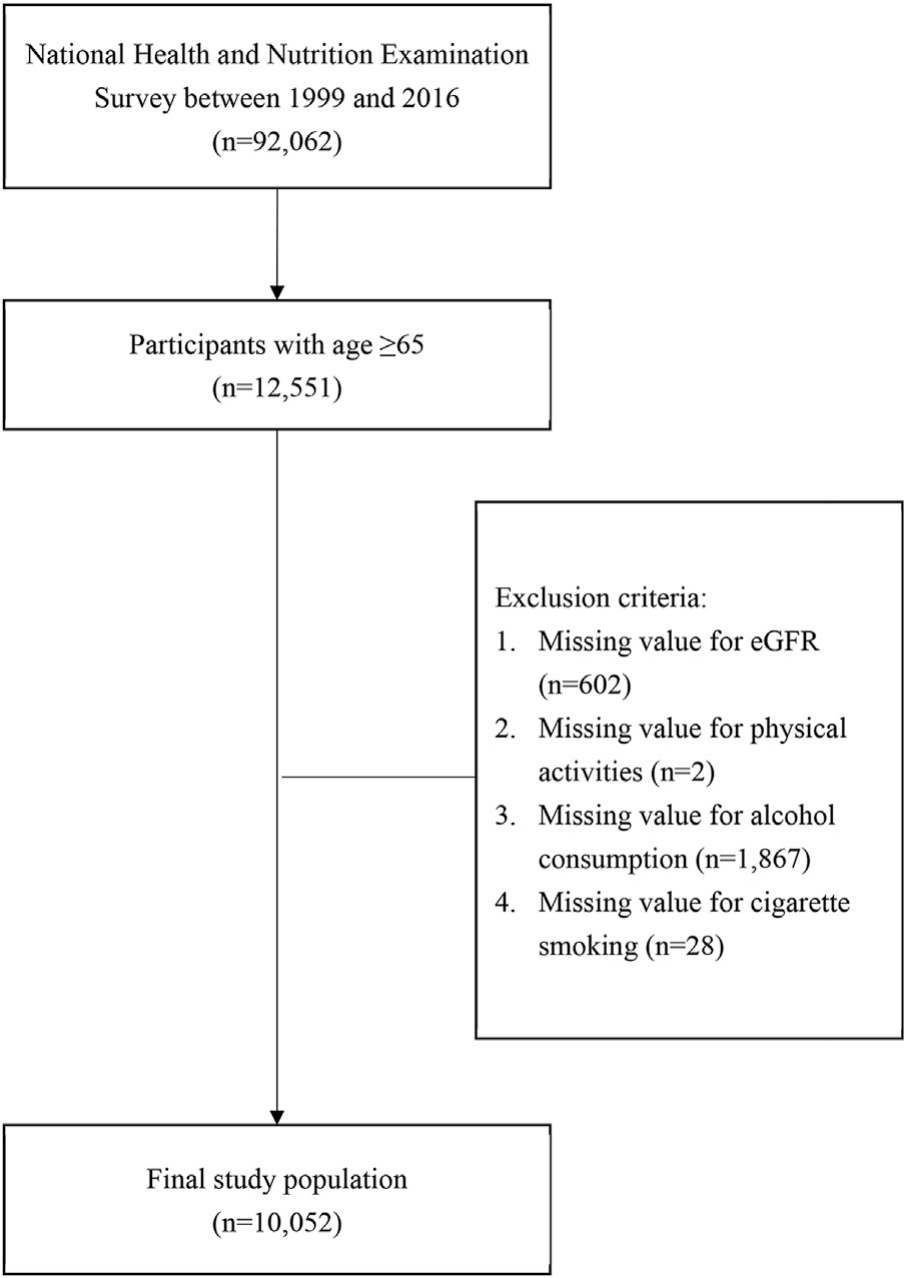
Flowchart of study sample inclusion and exclusion criteria. (Associations between Lifestyle Factors and Reduced Kidney Function in US Older Adults: NHANES 1999-2016).

A total of 92,062 participants were included in the NHANES database from 1999 to 2016, of whom 12,551 participants were aged 65 years or older (Figure 1). After excluding participants with missing values for eGFR and/or missing data for physical activity, alcohol consumption, or cigarette smoking, 10,052 participants were included as the analytic sample in the present study (Figure 1).

Eligible participants were stratified into two groups by eGFR level: the normal eGFR group (eGFR ≥ 60 ml/min/1.73 m^2^, *n* = 6,951) and the reduced eGFR group (eGFR < 60 ml/min/1.73 m^2^, *n* = 3,101). The demographic, lifestyle-related, and clinical characteristics of the two eGFR groups are shown in Table 1. The mean ages were 72.27 and 75.77 years for participants with normal eGFR and reduced eGFR, respectively. The percentages of participants aged 65–74 years were 66.23 and 40.28% in the normal and reduced eGFR groups, respectively. In addition to age, sex, race/ethnicity, education level, poverty/income ratio, BMI, physical activity, alcohol consumption, and cigarette smoking were significantly different between the two eGFR groups (all *p* ≤ 0.0001; Table 1). Compared to the normal eGFR group, the reduced eGFR group had significantly higher blood levels of fasting glucose, total cholesterol, uric acid, BUN, and creatinine as well as a significantly higher percentage of proteinuria (all *p* ≤ 0.02). However, the HDL blood levels were significantly lower in the reduced eGFR group than those in the normal eGFR group (*p* = 0.0005). Regarding comorbidities, the reduced eGFR group has significantly higher percentages of cardiovascular disease, diabetes, hyperuricemia, hypertension, and cancer than the normal eGFR group (all *p* ≤ 0.002); however, the prevalence of hyperlipidemia was significantly higher in the normal eGFR group (*p* = 0.04; Table 1).

**TABLE 1:** Demographic, lifestyle-related, and clinical characteristics of the study population stratified by eGFR levels. (Associations between Lifestyle Factors and Reduced Kidney Function in US Older Adults: NHANES 1999-2016).

	eGFR ≥ 60	eGFR < 60	*p*-value
	*n* = 6,951	*n* = 3,101	
**Demographics**			
Age, years	72.27 ± 0.10	75.77 ± 0.14	< 0.0001^a^
65–74	4,357 (66.23%)	1,120 (40.28%)	< 0.0001^a^
75+	2,594 (33.77%)	1981 (59.72%)	
Sex			
Male	3,608 (46.48%)	1,443 (39.11%)	< 0.0001^a^
Female	3,343 (53.52%)	1,658 (60.89%)	
Race/ethnicity			
Mexican american	1,072 (3.87%)	264 (2.3%)	< 0.0001^a^
Other hispanic	500 (3.56%)	150 (2.57%)	
Non-hispanic white	3,875 (81.07%)	2,135 (85.09%)	
Non-hispanic black	1,159 (7.49%)	429 (6.62%)	
Other-including multi-racial	345 (4%)	123 (3.43%)	
Education level			
Without high school diploma	2,483 (23.23%)	1,107 (27.91%)	0.0001^a^
High school diploma	1,610 (25.60%)	803 (28.18%)	
Higher than high school	2,849 (51.08%)	1,183 (42.75%)	
Unknown/missing	9 (0.09%)	8 (0.16%)	
Poverty/income ratio			
<1	1,055 (8.97%)	447 (10.25%)	< 0.0001^a^
1–3	3,165 (41.87%)	1,544 (48.36%)	
>3	2070 (40.10%)	830 (33.03%)	
Unknown/missing	661 (9.07%)	280 (8.36%)	
BMI^b^			
Underweight	104 (1.62%)	29 (0.98%)	< 0.0001^a^
Normal	1847 (26.73%)	772 (25.57%)	
Overweight	2,647 (37.67%)	1,121 (35.61%)	
Obesity	2,194 (31.99%)	1,045 (34.21%)	
Unknown/missing	159 (1.98%)	134 (3.63%)	
**Lifestyle**			
Physical activity (MET)^c^	872.60 ± 30.53	582.29 ± 42.62	< 0.0001^a^
Inactive	3,262 (43.1%)	1823 (56.26%)	< 0.0001^a^
Insufficiently active	1,637 (24.77%)	640 (22.48%)	
Sufficiently active	2052 (32.14%)	638 (21.27%)	
Alcohol consumption			
No	1,288 (16.34%)	699 (22.71%)	< 0.0001^a^
Former drinker	2,464 (32.42%)	1,293 (38.3%)	
Current drinker	3,199 (51.24%)	1,109 (38.99%)	
Cigarette smoking			
No	3,301 (47.56%)	1,547 (51.6%)	< 0.0001^a^
Former smoker	2,890 (42.64%)	1,321 (41.69%)	
Current smoker	760 (9.81%)	233 (6.71%)	
Blood examination			
Fasting glucose, mg/dL	113.02 ± 0.77	116.35 ± 1.19	0.02^a^
Total cholesterol, mg/dL	119.05 ± 0.72	192.94 ± 1.06	< 0.0001^a^
HDL, mg/dL	55.12 ± 0.61	51.26 ± 0.77	0.0005^a^
Uric acid, mg/dL	5.35 ± 0.02	6.46 ± 0.04	< 0.0001^a^
BUN, mg/dL	15.04 ± 0.08	22.80 ± 0.20	< 0.0001^a^
Creatinine, mg/dL	0.85 ± 0.003	1.36 ± 0.02	< 0.0001^a^
Proteinuria^d^	1,422 (18.15%)	1,100 (31.42%)	< 0.0001^a^
Comorbidities			
Cardiovascular disease	1,564 (22.02%)	1,237 (38.54%)	< 0.0001^a^
Diabetes	1,402 (16.86%)	890 (26.31%)	< 0.0001^a^
Hyperlipidemia	5,253 (78.68%)	2,296 (76.34%)	0.04^a^
Hyperuricemia	1,018 (13.25%)	1,182 (36.09%)	< 0.0001^a^
Hypertension	4,530 (63.31%)	2,424 (77.51%)	< 0.0001^a^
Urinary tract stones^e^	523 (8.10%)	259 (8.67%)	0.45
Cancer	1,463 (25.13%)	800 (28.83%)	0.002^a^

Continuous variables are expressed as mean ± standard error (SE) and tested by Complex Samples General Linear Model, while categorical variables are presented as counts (weighted percentage) and tested by Rao-Scott Chi-square test.

eGFR, estimated glomerular filtration rate; BMI, body mass index; MET, metabolic equivalent; HDL, high density lipoprotein; BUN, blood urea nitrogen.

a Statistical significance *p* < 0.05.

b Underweight: BMI <18.5 kg/m^2^, normal: 18.5 ≤ BMI <25.0 kg/m^2^, overweight: 25.0 ≤ BMI <30.0 kg/m^2^, obesity: BMI ≥30 kg/m^2^.

c Physical activity was defined by MET scores. Participants were divided into three MET groups: Inactive: 0 MET-min/week; Insufficiently active: <750 MET-min/week; Sufficiently active: ≥750 MET-min/week.

d Urinary albumin-to-creatinine ratio >3 mg/mmol.

e The questionnaire used for defining kidney stones has been available since 2007 (*n* = 4,653).

The significant interaction terms between covariates and their associations with reduced kidney function are presented in Table 2. After adjusting for the significant variables identified in the univariate logistic analysis and the significant interaction terms, multivariable logistic analysis revealed that older age [adjusted odds ratio (aOR) = 2.87, 95% CI = 2.47–3.34), proteinuria (aOR = 1.63, 95% CI = 1.37–1.95), cardiovascular disease (aOR = 1.87, 95% CI = 1.55–2.27), diabetes (aOR = 1.66, 95 %CI = 1.33–2.08), hyperuricemia (aOR = 3.10, 95 %CI = 2.57–3.73), and hypertension (aOR = 1.38, 95% CI = 1.21–1.57) were significantly associated with higher odds of having decreased kidney function (Table 3). In contrast, more recent survey cycle (aOR = 0.97, 95%CI = 0.95–1.00), Mexican American (aOR = 0.44, 95%CI = 0.33–0.59), Other Hispanic (aOR = 0.66, 95% CI = 0.45–0.98), Non-Hispanic Black (aOR = 0.51, 95% CI = 0.42–0.62), Other Race (aOR = 0.70, 95% CI = 0.50–0.99), sufficient physical activity (aOR = 0.75, 95% CI = 0.64–0.87), current alcohol consumption (aOR = 0.63, 95% CI = 0.47–0.85), and being a current smoker (aOR = 0.76, 95% CI = 0.61–0.96) were significantly associated with a lower risk of having decreased kidney function (Table 3). Furthermore, the results of the sensitivity analysis were consistent with the results of the multivariable logistic analysis, except for sex and diabetes (Table 3).

**TABLE 2:** Significant interaction terms between covariates, and their associations with reduced kidney function. (Associations between Lifestyle Factors and Reduced Kidney Function in US Older Adults: NHANES 1999-2016).

**Interaction**	aOR (95% CI)
Age x cardiovascular disease	0.76 (0.58–1.00)
Age x diabetes	0.74 (0.55–0.98)
Female x former drinker	1.22 (0.89–1.68)
Female x current drinker	1.41 (1.01–1.98)
Female x proteinuria	0.72 (0.56–0.94)
Female x hyperuricemia	1.50 (1.15–1.96)
Mexican american x proteinuria	1.77 (1.21–2.60)
Other hispanic x proteinuria	1.58 (0.72–3.45)
Non-hispanic black x proteinuria	1.69 (1.23–2.30)
Other racial x proteinuria	1.14 (0.58–2.27)

Multivariate model was adjusted for age, sex, race, physical activity, alcohol consumption, cigarette smoking, proteinuria, cardiovascular disease, diabetes, hyperlipidemia, hyperuricemia, hypertension, cancer, and interaction terms.

**TABLE 3:** Associations between risk factors and reduced kidney function (eGFR < 60 ml/min/1.73 m^2^) evaluated by logistic regression analysis. (Associations between Lifestyle Factors and Reduced Kidney Function in US Older Adults: NHANES 1999-2016).

	Univariate	Multivariate^c^	
	Or (95%CI)	aOR (95% CI)	β ± SE
Survey cycle	**0.97 (0.95–1.00)**	**0.97 (0.95–1.00)**	**0.41** ± **0.11**
**Demographics**			
Age, years			
65–74	Reference	Reference	Reference
75+	**2.91 (2.61–3.24)**	**2.87 (2.47–3.34)**	−**6.73** ± **0.56**
Sex			
Male	Reference	Reference	Reference
Female	**1.35 (1.21–1.51)**	1.25 (0.92–1.69)	−**1.37** ± **1.07**
Race/ethnicity			
Mexican american	**0.57 (0.48–0.67)**	**0.44 (0.33–0.59)**	**8.33** ± **0.96**
Other hispanic	**0.69 (0.53–0.89)**	**0.66 (0.45–0.98)**	**3.79** ± **1.28**
Non-hispanic white	Reference	Reference	Reference
Non-hispanic black	**0.84 (0.74–0.96)**	**0.51 (0.42–0.62)**	**8.24** ± **0.79**
Other - including multi-racial	0.82 (0.64–1.04)	**0.70 (0.50–0.99)**	**3.14** ± **1.08**
Education level			
Without high school diploma	Reference	Reference	Reference
High school diploma	0.92 (0.80–1.05)	0.99 (0.85–1.17)	−0.73 ± 0.64
Higher than high school	**0.71 (0.62–0.82)**	0.94 (0.80–1.10)	−0.16 ± 0.65
Unknown/missing	1.59 (0.52–4.88)	2.01 (0.64–6.35)	−6.12 ± 4.03
Poverty/income ratio			
<1	Reference	Reference	Reference
1–3	1.01 (0.86–1.18)	0.99 (0.82–1.19)	−1.23 ± 0.76
>3	**0.72 (0.60–0.86)**	1.05 (0.85–1.29)	−1.12 ± 0.65
Unknown/missing	**0.81 (0.65–1.00)**	0.91 (0.73–1.14)	−0.16 ± 0.65
BMI			
Underweight	0.63 (0.37–1.09)		
Normal	Reference	-	-
Overweight	0.99 (0.87–1.13)		
Obesity	1.12 (0.98–1.27)		
Unknown/missing	**1.91 (1.44–2.54)**		
Lifestyle			
Physical activity			
Inactive	Reference	Reference	Reference
Insufficiently active	**0.70 (0.60–0.80)**	0.86 (0.75–1.00)	0.06 ± 0.53
Sufficiently active	**0.51 (0.44–0.59)**	**0.75 (0.64–0.87)**	**1.12 ± 0.54**
Alcohol consumption			
No	Reference	Reference	Reference
Former drinker	**0.85 (0.74–0.97)**	0.79 (0.60–1.05)	0.87 ± 1.04
Current drinker	**0.55 (0.47–0.64)**	**0.63 (0.47–0.85)**	**3.29 ± 1.03**
Cigarette smoking			
No	Reference	Reference	Reference
Former smoker	0.90 (0.80–1.01)	0.88 (0.76–1.00)	**1.77 ± 0.54**
Current smoker	**0.63 (0.51–0.78)**	**0.76 (0.61–0.96)**	**3.57 ± 0.94**
Blood examination			
Proteinuria^a^	**2.16 (1.92–2.42)**	**1.63 (1.37–1.95)**	−**4.68 ± 0.77**
Comorbidities			
Cardiovascular disease	**2.22 (1.99–2.48)**	**1.87 (1.55–2.27)**	−**4.89 ± 0.80**
Diabetes	**1.76 (1.50–2.06)**	**1.66 (1.33–2.08)**	−1.39 ± 0.74
Hyperlipidemia	**0.87 (0.77–0.99)**	0.91 (0.78–1.05)	0.04 ± 0.51
Hyperuricemia	**3.70 (3.27–4.18)**	**3.10 (2.57–3.73)**	−**9.69 ± 0.71**
Hypertension	**2.00 (1.77–2.25)**	**1.38 (1.21–1.57)**	−**2.65 ± 0.50**
Urinary tract stones^b^	1.08 (0.89–1.30)	–	–
Cancer	**1.21 (1.07–1.36)**	1.09 (0.95–1.25)	−0.36 ± 0.54

OR: odds ratio; aOR: adjusted odds ratio; CI: confidence interval; eGFR, estimated glomerular filtration rate; BMI, body mass index.

Significant values are in **bold** (*p* < 0.05).

a Urinary albumin-to-creatinine ratio >3 mg/mmol.

b The questionnaire used for defining kidney stones has been available since 2007 (*n* = 4,653).

c Multivariate model was adjusted for age, sex, race, physical activity, alcohol consumption, cigarette smoking, proteinuria, cardiovascular disease, diabetes, hyperlipidemia, hyperuricemia, hypertension, cancer, and significant interaction terms.

**FIGURE 2:**
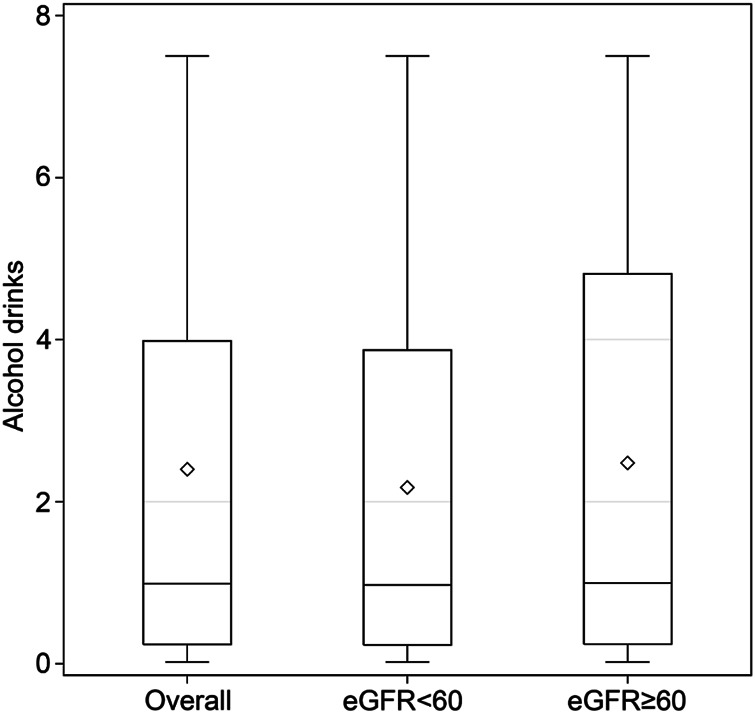
Number of alcoholic drinks per week in participants with current alcohol consumption. There were 4,308 current drinkers, including 1,109 with eGFR < 60 ml/min/1.73 m^2^ and 3,199 with eGFR ≥ 60 ml/min/1.73 m^2^. Data are displayed as minimum, first quartile, third quartile, and maximum with a *line* at the median value and a *diamond* at the mean value in the box-and-whisker plot. (Associations between Lifestyle Factors and Reduced Kidney Function in US Older Adults: NHANES 1999-2016).

The distribution of alcohol drinks per week in participants with current alcohol consumption, including all current drinkers, those with reduced eGFR, and those with normal eGFR, is shown in Figure 2. The median (IQR) values were 0.97 (0.23–3.87) and 0.99 (0.24–4.81) for current alcohol drinkers with reduced eGFR and with normal eGFR, respectively (Figure 2).

After stratifying by sex, the associations between the covariates and reduced kidney function were evaluated (Table 4). Multivariable models were adjusted for significant variables identified in the univariate analyses and the significant interaction terms, but sex-related interaction terms were excluded in this subgroup analysis. The results revealed that males of older age (aOR = 2.88, 95% CI = 2.32–3.58), proteinuria (aOR = 1.68, 95% CI = 1.39–2.04), cardiovascular disease (aOR = 1.91, 95%CI = 1.49–2.45), diabetes (aOR = 1.64, 95% CI = 1.21–2.22), hyperuricemia (aOR = 3.12, 95% CI = 2.61–3.81), and hypertension (aOR = 1.36, 95% CI = 1.13–1.61) were significantly associated with higher odds of decreased kidney function. In contrast, males with current alcohol consumption (aOR = 0.63, 95% CI = 0.46–0.85) had significant lower odds of decreased kidney function (Table 4). On the other hand, females of older age (aOR = 2.89, 95% CI = 2.33–3.57), cardiovascular disease (aOR = 1.83, 95% CI = 1.35–2.49), diabetes (aOR = 1.69, 95% CI = 1.27–2.25), and hyperuricemia (aOR = 4.63, 95% CI = 3.83–5.60) were significantly associated with higher odds of decreased kidney function. But females with sufficient physical activity (aOR = 0.66, 95% CI = 0.53–0.82) had significant lower odds of decreased kidney function (Table 4).

**TABLE 4:** Associations between risk factors and reduced kidney function (eGFR < 60 ml/min/1.73 m^2^) by sex. (Associations between Lifestyle Factors and Reduced Kidney Function in US Older Adults: NHANES 1999-2016).

	Male	Female
	aOR (95%CI)	aOR (95%CI)
Survey cycle	0.97 (0.94–1.01)	0.97 (0.94–1.01)
**Demographics**		
Age, years		
65–74	Reference	Reference
75+	**2.88 (2.32–3.58)**	**2.89 (2.33–3.57)**
Race/ethnicity		
Mexican american	**0.55 (0.39–0.77)**	**0.38 (0.25–0.58)**
Other hispanic	0.84 (0.54–1.32)	**0.58 (0.35–0.97)**
Non-hispanic white	Reference	Reference
Non-hispanic black	**0.50 (0.37–0.69)**	**0.53 (0.40–0.69)**
Other-including multi-racial	**0.52 (0.29–0.94)**	0.87 (0.54–1.41)
Education level		
Without high school diploma	Reference	Reference
High school diploma	0.81 (0.63–1.04)	1.13 (0.91–1.41)
Higher than high school	0.85 (0.67–1.08)	1.00 (0.81–1.22)
Unknown/missing	1.57 (0.37–6.75)	2.38 (0.50–11.44)
Poverty/income ratio		
< 1	Reference	Reference
1–3	0.91 (0.70–1.19)	1.03 (0.81–1.32)
>3	1.01 (0.73–1.40)	1.06 (0.80–1.39)
Unknown/missing	0.93 (0.65–1.33)	0.90 (0.65–1.23)
Lifestyle		
Physical activity		
Inactive	Reference	Reference
Insufficiently active	0.92 (0.73–1.15)	0.83 (0.69–1.00)
Sufficiently active	0.86 (0.69–1.05)	**0.66 (0.53–0.82)**
Alcohol consumption		
No	Reference	Reference
Former drinker	0.80 (0.60–1.08)	0.96 (0.80–1.15)
Current drinker	**0.63 (0.46–0.85)**	0.89 (0.71–1.11)
Cigarette smoking		
No	Reference	Reference
Former smoker	0.87 (0.72–1.04)	0.88 (0.74–1.05)
Current smoker	0.70 (0.47–1.03)	0.83 (0.62–1.11)
Blood examination		
Proteinuria^a^	**1.68 (1.39–2.04)**	1.16 (0.93–1.45)
Comorbidities		
Cardiovascular disease	**1.91 (1.49–2.45)**	**1.83 (1.35–2.49)**
Diabetes	**1.64 (1.21–2.22)**	**1.69 (1.27–2.25)**
Hyperlipidemia	0.89 (0.73–1.07)	0.93 (0.75–1.15)
Hyperuricemia	**3.12 (2.61–3.81)**	**4.63 (3.83–5.60)**
Hypertension	**1.36 (1.13–1.61)**	**1.39 (1.15–1.69)**
Cancer	1.02 (0.85–1.22)	1.15 (0.94–1.40)

OR: odds ratio; aOR: adjusted odds ratio; CI: confidence interval; eGFR, estimated glomerular filtration rate.

Significant values are in **bold** (*p* < 0.05).

a Urinary albumin-to-creatinine ratio >3 mg/mmol.

b Multivariate model was adjusted for survey cycle, age, sex, race, physical activity, alcohol consumption, cigarette smoking, proteinuria, cardiovascular disease, diabetes, hyperlipidemia, hyperuricemia, hypertension, cancer, and significant interaction terms (except for sex-related interaction terms).

## Discussion

The main findings of the present cross-sectional study conducted among older adults in the United States NHANES database were that older age, proteinuria, cardiovascular disease, diabetes, hyperuricemia, and hypertension were significantly associated with higher odds of reduced kidney function, while sufficient physical activity, current alcohol consumption, and being a current smoker were significantly associated with lower odds of reduced kidney function. Furthermore, subgroup analysis by sex revealed several sex-differential associations. Firstly, proteinuria was a risk factor for reduced eGFR in males only. In contrast, sufficient physical activity served as a beneficial factor only in female participants, and only males could be benefited from current alcohol consumption. Also, the beneficial effect of being a current smoker was no longer significant in males or females.

The results of previous studies conducted in other populations report similar findings about the impact of exercise, alcohol consumption, smoking, and sex on the eGFR levels representing reduced kidney function in older adults. An elderly population-based study found that, among individuals with eGFR < 60, only 3.5% were without renal damage or comorbidities associated with a risk of renal insufficiency, and those with eGFR > 60 had none of the associated risk factors for reduced kidney function [13]. The authors of that study also suggest that, although the eGFR in older adults has long been associated with aging itself, and that a low eGFR is also associated with incident frailty in the elderly population [14], the eGFR appears to be directly associated with CKD and comorbidities such as cardiovascular disease and hypertension [13]. Perhaps instead of screening for CKD using eGFR as an indicator of reduced kidney function, it may make more sense clinically to assess vascular health, which is shown to increase the risk of reduced kidney function in older adults [15].

Investigation of the Impact Goals of the American Health Association (AHA), which were developed as part of cardiovascular health promotion, for associations with risk of incident CKD showed that the AHA’s “Life’s Simple 7” promoting cardiovascular health simultaneously predicts a lower risk of CKD [16]. The Simple 7 factors are: nonsmoker or quit >1 year ago; BMI < 25 kg/m^2^; ≥150 min/week of physical activity; healthy dietary habits; total cholesterol < 200 mg/dl; blood pressure < 120/80 mmHg; and fasting blood glucose < 100 mg/dl [16]. Smoking was also recently shown to be associated with a higher risk of developing CKD in healthy middle-aged Korean adults [17]. When the authors stratified their study population by age (<60 vs. >60 years), sex, smoking, diabetes, hypertension, and BMI, no significant interactions were found between the factors and the effects of current smoking on CKD [17].

In the present study, among the participants with current alcohol consumption, the majority were shown to consume four or fewer alcohol-based drinks per week, suggesting that mild to moderate alcohol was consumed by older participants. On the other hand, most participants with normal eGFR had five or fewer alcohol-based drinks per week. Importantly, the maximum number of alcohol drinks per week was less than eight in the present study, so it may be reasonable to assume that the included older adult participants consumed mild to moderate amounts of alcohol per week currently. In previous studies in the United States and Japan, mild to moderate alcohol consumption has been suggested to be associated with a lower risk of developing CKD [18, 19]. Hu et al. [18] conducted a prospective study of over 12,000 subjects in six alcohol consumption categories, finding, as we did, that drinking a moderate amount of alcohol may not harm the kidneys and is not associated with CKD based on an eGFR of < 60 ml/min/1.73 m^2^. Matsumoto et al. [19] looked at both smoking and alcohol as risk factors contributing to CKD, finding that mild to moderate alcohol consumption may be associated with CKD (i.e., low eGFR and proteinuria), but the same association was not shown in smokers; they concluded that the risk of CKD may be less in smokers because smoking modifies the potential beneficial effect of alcohol in preventing CKD. The differences between the studies may be due to the different age groups studied.

In our previous study in a large Taiwanese population [10], male sex was significantly associated with an increased odds of having a reduced eGFR. Although the present NHANES study found that female older adults were about 1.25 times more likely to have reduced kidney function than are their male counterparts, such an association did not reach a significant level. In seeking to explain the gender discrepancies between that study and the present study, we stratified all participants by sex and re-evaluated the associations between life factors and reduced kidney function. Almost all of the risk factors identified in the population were still valid in either male or female participants, except proteinuria that was significant in males only. In addition, differences in the beneficial factors between males and females were also observed. Males and females benefited from current alcohol consumption and sufficient physical activity, respectively. Surprisingly, after stratifying by sex, being a current smoker was no longer a beneficial factor for reduced kidney function in males and females. The differences between the studies may be partially explained, as in Carrero et al. [20], by the greater prevalence of CKD in women and the tendency of longer life among women; thus, in study populations, we must consider the effects of longer life on natural declines in the eGFR. The authors also pointed out the potential overdiagnosis of CKD through the inappropriate use of eGFR equations [20]. Akasaka et al. [21] also emphasized the influence of elevated uric acid levels, characteristic of a reduced kidney function, on both eGFR and gender differences; uric acid concentration appears to accelerate the decline in eGFR values, and women appear to be more susceptible to urate-induced eGFR decline.

In the present study, physical exercise as reported in the NHANES database was measured in METs, which converts frequency and duration to minutes of activity per week, weighted by multiplying by an estimated MET value using the Compendium of Physical Activity values for intensity assessment [15] and summed across all activities to total physical activity. The MET results showed that participants with sufficient physical activity had significantly lower odds of having decreased kidney function. In our previous cross-sectional study [10], which analyzed data from the Taipei City Elderly Health Examination database 2006–2012, the results showed that participants who had regular or occasional exercise had significantly lower risks of having reduced kidney function than those without exercise.

The effects of home-based exercise therapy on kidney function in pre-dialysis CKD patients have also been explored previously [22], demonstrating that home-based exercise therapy *did not* affect kidney function in pre-dialysis CKD patients, even though it helped to improve leg muscle strength. A study that examined the eGFR levels in community-based older adults after a 12-week exercise program and post-exercise protein supplementation had modest positive effects and did not show a deleterious effect on kidney function, but the endpoint eGFR levels of the combined intervention were not significantly different before and after [23]. A cross-sectional study of the association between physical activity and kidney function in older men reported that higher levels of physical activity were associated with reduced odds of a lower eGFR, while each added 30 min of sedentary behavior per day was associated with greater odds of a lower eGFR [24]. Even without knowing whether the associations are causal, they were projected to be of public health concern in men with other risk factors for CKD.

### Strengths and Limitations

The present study was strengthened by the use of data from nine 2-year cycles of the NHANES database 1999–2016, a continuous cross-sectional health survey that collects and analyzes data representative of the national United States non-institutionalized population. Because we included over 10,000 older adults with eGFR values, the sample size is large enough for fairly precise prevalence measures at the national level. Conducting this study using a nationally representative sample allows our results to be generalized across the entire United States older adult population. Nevertheless, certain limitations must also be noted, including that it involved cross-sectional analysis, which precludes making inferences about causality. In addition, the NHANES data are from interviews and self-reported questionnaires and may be subject to recall problems or misunderstanding of the questions, which may reduce the accuracy of the data to some extent. The NHANES database also includes only United States data, including representative portions of different ethnic populations, and may not support conclusions about racial/ethnic factors or any populations outside of the United States. Further studies are needed to validate these findings in other populations and countries.

### Conclusion

The present study identified several risk and beneficial factors for reduced kidney function in adults aged 65 and above and found that some risk and beneficial factors might be sex-specific. These findings may be useful in developing strategies for public health policies regarding risk and beneficial factors associated with reduced kidney function in older adults, as well as helping to explore whether screening using eGFR may be of value toward the early diagnosis of reduced kidney function in high-risk older adult populations.
